# Supplementary material for: Receptor Transporter Protein 4 (RTP4) in the Hypothalamus Is Involved in the Development of Antinociceptive Tolerance to Morphine
Source: Biomolecules. 2022 Oct 13;12(10):1471. doi: 10.3390/biom12101471 (PMC9599210; doi:10.3390/biom12101471)
Supplement: Supplementary file 1 [file biomolecules-12-01471-s001.zip › biomolecules-1891322-supplementary.pdf]

Supplementary Figure S1

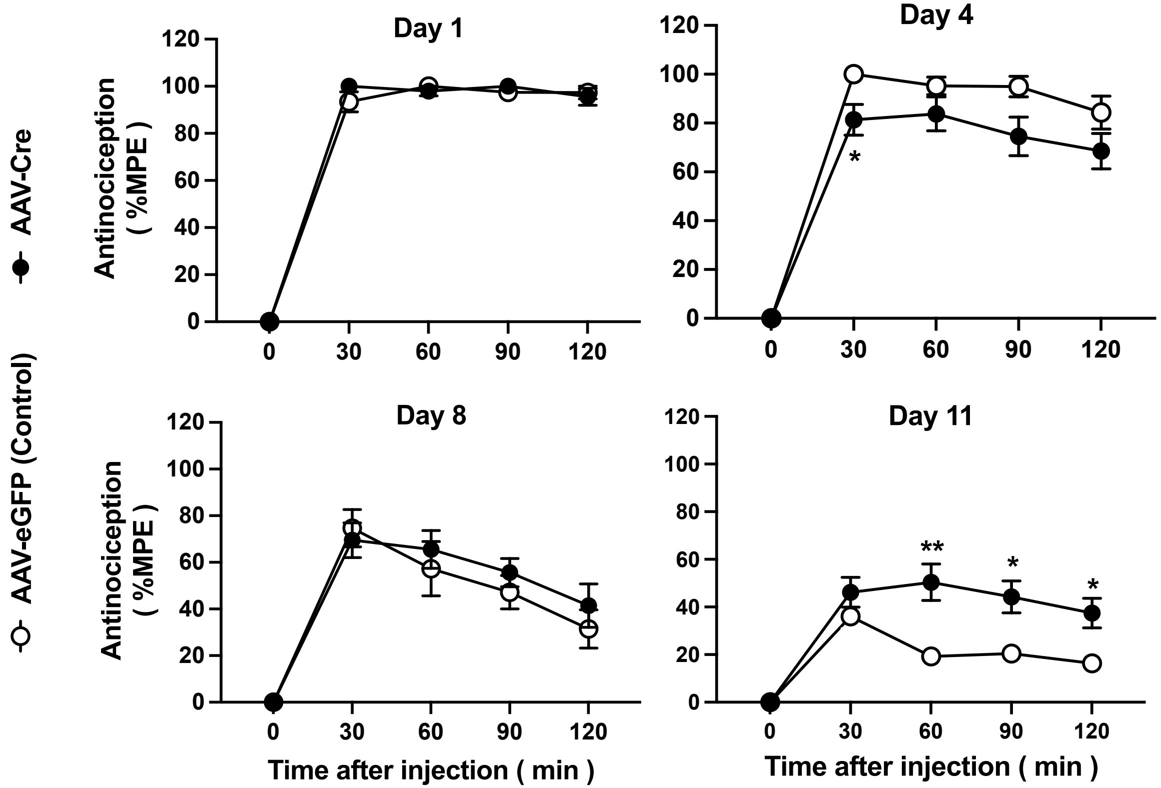

Figure S1. The effect of knockdown of RTP4 in PVN on the development of antinociceptive tolerance to morphine. The antinociceptive effect of morphine after morphine administration on Day 1, Day4, Day8 and Day 11. Data are the mean ± S.E.M. n=7 (AAV-eGFP, control, open circle), n=10 (AAV-Cre, closed circle), *p<0.05, **p<0.01, vs. AAV-eGFP (Control), unpaired t-test.

Supplementary Table S1

Molecules	Relative mRNA expression (GAPDH = 1000)	n
TLR4	2.538 ± 0.079	6
OPRM1	0.013 ± 0.002	12
OPRD1	0.002 ± 0.0001	12
RTP4	0.339 ± 0.031	12

Table S1. The relative expression of TLR4, OPRM1, OPRD1 and RTP4 mRNA in SIM-A9 cells without any treatment. RT-qPCR was performed with specific primers against TLR4, OPRM1, OPRD1 and RTP4. Data are the mean ± S.E.M. n=6-12
